# Special Issue “Recent Research on Natural Products from Microalgae and Cyanobacteria”

**DOI:** 10.3390/ijms27135833

**Published:** 2026-06-28

**Authors:** Biswajita Pradhan

**Affiliations:** 1 Department of Biotechnology, Sangmyung University, Seoul 03016, Republic of Korea; pradhan.biswajita2014@gmail.com; Tel.: +91-8260018085; 2 Department of Botany, Model Degree College, Rayagada 765017, Odisha, India

Cyanobacteria and microalgae are among the most underexplored sources of bioactive natural compounds [1]. Due to their ecological significance, metabolic diversity, and prospective applications in biotechnology, medicine, nutraceuticals, and cosmetics, these photosynthetic organisms have garnered a lot of interest in recent decades [2,3]. Recent research has revealed remarkable advances in metabolomics and high-throughput screening, which have led to the discovery of new peptides, alkaloids, and polyketides with antioxidant, antimicrobial, anticancer, antidiabetic, anti-inflammatory, and antiviral properties [4,5,6]. Microalgae-derived pigments (e.g., phycocyanin, astaxanthin) and polyunsaturated fatty acids are increasingly used in food supplements and functional foods [7]. This Special Issue compiles state-of-the-art research on natural products derived from microalgae and cyanobacteria that demonstrates the potential use of these organisms for human benefit (see the List of Contributions for details).

In addition to addressing the ethical issues and contamination threats related to fetal bovine serum (FBS), the manufacture of serum-free media (SFM) is essential to the advancement of cell-culture techniques used in the production of viral vaccines [8]. In this regard, Park et al. created serum-free culture systems using marine microalgal extracts for Vero and Madin–Darby canine kidney (MDCK) cells (contribution 1). Park et al. also found that marine microalgal species extracts like *Dunaliella salina* (DS) and *Spirulina platensis* (SP) significantly increased the proliferation rate of both MDCK and Vero cells, with proliferation rates of 149.56% and 195.50% in MDCK and Vero cells, compared to serum-free medium (SFM), and in comparison to *Haematococcus pluvialis* (HP), *Nannochloropsis salina* (NS), and *Tetraselmis* sp. (TS). Moreover, superoxide dismutase (SOD) activity was notably elevated by DS and SP extracts (118.61% and 108.72% in MDCK cells, and 130.08% and 125.63% in Vero cells), suggesting reduced intracellular oxidative stress.

Cyanobacteria are a possible biological platform for the production of biochemicals from CO_2_, such as carboxylic acids and their derivatives [9]. You et al. considered the TCA cycle and cyanobacterial acetyl-CoA pool as a significant source of precursor molecules for the biosynthesis of carboxylic acids, including 3-hydroxypropionate, 3-hydroxybutyrate, succinate, malate, fumarate, and free fatty acids, each of which is a key platform chemical for the bioeconomy (contribution 2). Additionally, they emphasized the unique characteristics of the cyanobacterial TCA cycle, how it differs from the well-documented TCA cycles of heterotrophic model organisms in terms of structure and function, and how to improve its suitability for synthesizing carboxylic acids from CO_2_. In order to make cyanobacteria viable for the large-scale, sustained synthesis of carboxylic acids and their derivatives, more research is required to improve their metabolic efficiency.

Paraskevopoulou et al. identified 31 significant odorants in *Spirulina* (*Arthrospira platensis*), of which 14 were reported as constituents for the first time using aroma extract dilution analysis and offline and online fractionation techniques (contribution 3). This will serve as a foundation for future odor optimization of *Spirulina*-based food products.

Improving the biomass yield and C-phycocyanin (C-PC) productivity of *Spirulina* sp., a cyanobacterium with numerous applications in the food, pharmaceutical, and biotechnological industries, requires optimizing nutritional formulations [10,11]. Nurjannah et al. used a two-level complete factorial design to compare eight Zarrouk-based formulations to the standard medium in order to assess the impact of macronutrient alterations on growth and pigment production. The cultivation of *Spirulina* sp. with the highest biomass output was F2 (16 g/L NaHCO_3_, 5 g/L NaNO3, 0.25 g/L K_2_HPO_4_), which produced a 37.6% increase in dry weight productivity compared to the control, and F3 (16 g/L Na-HCO_3_, 5 g/L NaNO_3_, 1 g/L K_2_HPO_4_) (contribution 4). Furthermore, the findings offer a logical framework for creating nutrient-efficient farming methods to promote sustainable biomanufacturing based on *Spirulina*.

Cyanobacteria are known for their varied and powerful bioactive compounds, which offer a novel approach to drug delivery because of their extracellular vesicles (EVs), which resemble exosomes in size and function but differ in biogenesis [12]. EVs are drawing interest as possible vehicles for targeted drug delivery because of their biocompatibility, stability, and capacity to encapsulate a variety of bioactive substances [13]. The potential therapeutic applications of cyanobacterial EVs have been examined with a focus on their involvement in immunological regulation, neuroprotection, antimicrobial treatments, and cancer treatment [12]. Asseri et al. described their biogenesis and structural characteristics, comparing them with artificial nanocarriers such as liposomes and polymeric nanoparticles (contribution 5). The unique properties of cyanobacterial EVs offer great promise for improving drug delivery systems and offering novel solutions for treating complex diseases, despite the difficulties in isolating and characterizing them at scale and the need for developments in synthetic biology and genetic engineering to optimize their therapeutic potential.

The production of many secondary metabolites contributes to the amazing resilience of microalgae to hostile conditions. Mycosporine-like amino acids (MAAs), which are low-molecular-weight, water-soluble, UV-absorbing substances with anti-inflammatory, anticancer, and antimicrobial properties, are known to be produced by cyanobacteria [14,15,16]. In contrast, under standard conditions, green microalgae usually do not exhibit detectable MAAs, and little is known about how they respond to abiotic stress [17]. Tsintzou et al. studied the freshwater green microalga *Jaagichlorella luteoviridis* cultivated under three stressors, namely heat, salinity, and UV, and evaluated MAA induction. Moreover, they optimized an MAA extraction protocol appropriate for “green” downstream applications in the pharmaceutical, nutraceutical, and cosmeceutical industries (contribution 6). Additionally, transcriptomic profiling against a reference genome showed stress-specific differential gene expression and overexpression of certain MAA pathway genes, such as ArioC and AroM/Aro1 SAM methyltransferases, identifying potential targets for engineering enhanced MAA production. This aligns with the market demand for safe and environmentally friendly bioactives; microalgae represent a promising source of these valuable molecules.

The phycobiliprotein phycocyanin has a potent anti-aging effect [18] and accounts for up to 20% of the dry weight of *Arthrospira platensis*, significantly prolonging the chronological life span (CLS) of yeast cells cultivated in synthetic-defined medium under both caloric restriction (CR) conditions (0.2% glucose) and non-CR conditions (2% glucose). Cassamagnaghi et al. found that while the Ras2/PKA route accelerates aging (pro-aging) in response to phycocyanin exposure, the Snf1 pathway promotes longevity (anti-aging) (contribution 7). Phycocyanin accelerated the chronological aging process of yeast cells, significantly reducing the CLS when glucose concentrations were low (0.2%) or high (2%) in YPDA-rich medium, suggesting that *Saccharomyces cerevisiae* could serve as a model organism to study the anti-aging characteristics and targets of phycocyanin, as well as its potential side effects, which may exist in higher eukaryotes under specific circumstances.

A number of exciting research directions are emerging, including metabolic engineering and synthetic biology, which could lead to the creation of customized strains that overproduce particular bioactive compounds; AI-driven discovery utilizing machine learning for direct compound identification and the anticipation of biosynthetic gene clusters; investigating extreme conditions and isolating new strains from unusual habitats (such as hot springs and polar locations) that may contain previously unknown metabolites; maximizing economic viability by incorporating holistic biorefinery principles, including multiple product streams like biofuels, nutraceuticals, and pharmaceuticals; and the validation of therapeutic potential through clinical translation and progression from preclinical research to human trials.

At the forefront of natural product research, microalgae and cyanobacteria have enormous potential for innovation in industry, sustainability, and health. This Special Issue offers a platform for advancing the field by combining new findings and addressing persistent challenges. We hope it encourages scientists to push boundaries, investigate unexplored metabolic landscapes, and translate discoveries into practical advantages for society in the future.
